# Management of respiratory tract infections in young children—A qualitative study of primary care providers’ perspectives

**DOI:** 10.1038/s41533-017-0018-x

**Published:** 2017-03-07

**Authors:** Ruby Biezen, Bianca Brijnath, Danilla Grando, Danielle Mazza

**Affiliations:** 1 Department of General Practice, Monash University, Building 1, 270 Ferntree Gully Road, Notting Hill, VIC 3168 Australia; 2 School of Occupational Therapy and Social Work, Curtin University, Building 401, Kent Street, Bentley, WA 6152 Australia; 3 School of Applied Sciences, RMIT University, Building 223, Level 1, Bundoora Campus, Plenty Road, Bundoora, VIC 3083 Australia

## Abstract

Respiratory tract infections in young children are the most common cause of general practice visits in Australia. Despite the availability of clinical practice guidelines, the treatment and management of respiratory tract infections in young children is inconsistent. The aim of the study was to explore the management of respiratory tract infections in young children from a multi-disciplinary perspective using across-sectional qualitative research design based on the theoretical domains framework and the Capability, Opportunity and Motivation-B model. In-depth interviews were conducted with 30 primary care providers to explore their knowledge, views and management of respiratory tract infections in young children. Interviews focused on symptomatic management, over-the-counter medications and antibiotic use, and data were thematically analysed. Our findings showed that factors such as primary care providers’ time constraints, parental anxiety, general practitioners’ perception of what parents want, perceived parental pressure, and fear of losing patients were some of the reasons why primary care providers did not always adhere to guideline recommendations. Primary care providers also provided conflicting advice to parents concerning over-the-counter medications and when children should resume normal activities. Overall, this study showed that complex interactions involving emotional and psychological factors influenced the decision making process of primary care providers’ management of respiratory tract infections in young children. A team care approach with consistent advice, and improved communication between primary care providers and parents is vital to overcome some of these barriers and improve guideline adherence. The findings of this research will inform the development of interventions to better manage respiratory tract infections in young children.

## Respiratory tract infections: Clinicians swayed by parental anxiety and pressure

The emotions and psychology of both parents and clinicians influence how respiratory tract infections (RTIs) are managed in young children. Researchers in Australia, led by Ruby Biezen from Monash University, interviewed 30 primary care clinicians about their views on how to care for children with RTIs, such as the common cold. The interviews focused on symptomatic management, over-the-counter medications and antibiotic use. Despite the availability of best-practice guidelines, clinicians did not always follow the recommendations owing to factors such as time constraints, parental anxiety, perceived parental pressure, and fear of losing patients. These are some of the reasons why clinicians sometimes advise or prescribe unnecessary medications. The authors suggest that a team approach involving multiple healthcare professionals who deliver consistent advice could improve guideline adherence.

## Introduction

Respiratory tract infections (RTIs) are the most frequent reason for general practice presentation in Australia.^1^ It is a major cause of morbidity, with young children (<5 years) being particularly vulnerable. Although the majority of RTIs are mild and self-limiting, the high prevalence of RTIs creates a significant health and economic burden,^2^ especially when carers’ time away from normal activities is taken into account.^3^ Australian clinical guidelines recommend fluid intake and rest for the treatment and management of most RTIs, and paracetamol can be given to children to reduce fever over 38.5 °C.^4, 5^ Oral decongestants and cough syrups are no longer recommended for children under the age of six due to the lack of evidence that they are effective and the possibility of side effects in this age group.^4–6^ As the majority of RTIs are caused by viruses, antibiotics are not warranted as a treatment for most RTIs.

Despite available clinical guidelines,^7^ general practitioners’ (GPs) treatment and management of RTIs in young children have been shown to be inconsistent in Australia; recent analysis of GP management of RTIs in children <5 years of age showed management varied widely according to the presenting clinical problem and the age and sex of the GP.^8^ Recent US studies have found that factors such as parental misconception regarding the symptoms of RTIs and their understanding of antibiotic use have influenced physicians’ management of RTIs in young children.^9, 10^ While Australian antibiotic use for RTIs in children compares favourably with overseas data,^8, 11, 12^ the rate of use is still higher than recommended guidelines.^4, 13^ Overseas studies have suggested antibiotic prescribing may be complicated by factors such as physicians’ diagnostic uncertainty, parents’ expectation of receiving antibiotics, physicians’ perception of what parents want, and parents’ satisfaction with the visit.^14–20^

In Australia, the reasons for the inconsistency with guideline recommendations in the management of RTIs in children <5 years of age, especially regarding antibiotic prescribing habits, are unclear. In addition, there have been no studies identified in the literature that have explored views concerning the management of RTIs in children from other primary care providers (PCPs) such as practice nurses (PNs), maternal child health nurses (MCHNs) and pharmacists. This group of professionals are a potentially valuable untapped resource as they are most likely to have more contact with the parents of children <5 years of age and would often provide advice to parents independently from the GP regarding the child’s health. By understanding the reasoning behind, the extent and how management of RTIs in young children differ from clinical guidelines, we may then be able to better manage RTIs in young children from both the parents and PCPs’ perspective. Therefore, the aim of our study is to explore the views, attitude and practices of PCPs such as GPs, PNs, MCHNs and pharmacists in the management of RTIs in young children.

## Results

Thirty PCPs including 20 GPs, two PNs, three MCHNs and five pharmacists participated in the study. Five major themes emerged from the PCP interviews as areas for change: (1) PCPs’ advice on managing RTIs in young children; (2) System barriers leading to lack of adherence to guidelines; (3) Parental anxiety affecting PCPs’ advice on treatment and management of RTIs; (4) Conflicting management advice between PCPs; and (5) Factors influencing GPs’ advice regarding antibiotic prescribing.

### Capability:Knowledge and skills

#### Theme 1: PCPs’ advice on managing RTIs in young children

All PCPs agreed that managing RTIs in young children should start with the management of symptoms, which includes rest, hydration, staying warm, and generally keeping the child comfortable. Paracetamol was advised if the child was irritable or uncomfortable or had a temperature above 38 °C. While some GPs recommended alternating paracetamol with ibuprofen in this age group, other GPs advised against using ibuprofen. Other ways of managing symptoms included saline nasal drops and vaporisers/steam inhalation to clear the nose. MCHNs also mentioned regular breastfeeding in a young baby to keep up fluids and advised going to see a GP if patients were generally unwell. Generally, PCPs commented that parents just wanted to be reassured that they were doing the right thing.

*“… a lot of the time they don’t necessarily need anything… a lot of them (parents) just say they want the reassurance that it’s a cold, so they are quite happy that they don’t necessarily want anything specific.” GP3.*

*“…most of them, most of the time … they just bring the kid in for reassurance…” GP7.*

PCPs recognised that other over-the-counter (OTC) medications such as decongestants and cough suppressants were no longer recommended for children under the age of 6 due to possible overdosing and/or sedation in this age group. Most GPs no longer recommended these OTC medications, however, some GPs mentioned that they did occasionally succumb to parental pressure:

*“…when the parents run in, they are driving me crazy… the nose doesn’t stop, they are coughing at night, then I might say, “look, there is not really good evidence that these things work… but you can try using them…” GP3.*

While GPs were hesitant recommending OTC medications in this age group, pharmacists turned to natural and complementary medicine such as Prospan (for chesty cough), Little Coughs (for coughs), Kaloba (for bronchitis and sinusitis) and Sambucol (cold and flu relief) in place of decongestants and cough suppressants. Although not proven to be clinically effective, pharmacists mentioned that these products were ‘‘all natural’’, ‘‘had antiviral benefits’’ and ‘‘built the immune system’’.

*“…so there’s a lot of things in my pharmacy such as the natural olive leaf extract, that I know I can give kids under 6…” PH2.*

*“…there are products now available on the market… something like Sambucol, they are black elderberries, so…yep, it’s natural…” PH3.*

### Opportunity: Social/environment

#### Theme 2: System barriers leading to lack of adherence to guidelines

System barriers such as the lack of time for PCPs to discuss management options for parents regarding RTIs, lack of opportunities to educate parents, and the pressure to perform led to non-adherence to clinical guidelines.

Lack of consultation time to educate parents was another reason why GPs diverted their actions from recommended guidelines. GPs commented that they were more likely to prescribe antibiotics if it was the last session on a Friday, or a Saturday morning; fatigue due to long consultation hours; running behind schedule with added parental pressure; and not being able to review the child until Monday. In addition, part time GPs were more likely to prescribe antibiotics as they had less opportunity to ask parents to come back for a review:

*“… the reality is, if it’s my last session, on a Friday, or on a Saturday morning, I might prescribe to them, but otherwise… I’ll try to educate them…” GP2*

“… *probably the part time general practitioners who might be here for a session or two a week, don’t have the luxury of a review within a short space of time… select to play it safe.” GP9*

In addition, GPs who practised in time constrained clinics would write a script rather than spend time to educate the parents.

*“Time constraints, you know, force GPs to dish them out.” GP14*

*“…I tend to prescribe more than it should be the case…” GP13*

Other PCPs thought GPs were generally time poor, however, they thought parents could obtain advice from other PCPs such as MCHNs and pharmacists as they are also trusted health-care professionals.

*“… through maternal child health nurses. I think that would be a good starting point, because … they are trusted health-care professionals, and just the fact that they have the time with the parents, the mother, whereas the GPs usually not…” PH3.*

### Motivation: Emotion

#### Theme 3: Parental anxiety affecting PCPs’ advice on treatment and management of RTIs

Parental anxiety coupled with the need to ‘‘do something’’ for the sick child was often mentioned as a key factor on PCPs’ decision to disregard guidelines and recommend medications that might not be needed. GPs recommended OTC medication and/or prescribed antibiotics to ‘‘please’’ anxious parents. Pharmacists said they sometimes faced pressure from parents to override the GP’s recommended medication treatment for their young child. Situations like this often made pharmacists feel uncomfortable.

*“… sometimes they (parents) come in and say that they are frustrated, they’ve just been to the doctor’s, doctor didn’t give them anything… they just want us to override the doctor’s advice, sell them something, so* … *we found ourselves in a position where we need to reinforce the message…“ PH3*

Most GPs recognised that parents’ anxiety could be minimised by reassuring anxious parents, explaining the nature of the fever, and writing down management plans so that parents felt like GPs were doing something for their sick child. A follow up review might be suggested if parents were overly anxious regarding their child’s cold symptoms. On other occasions, GPs said they would not disclose all the information to avoid increasing parents’ anxiety and parents’ expectation of treatment.

*“If you examine the child, sometimes you see the ears are a bit like pink or red, and if I don’t think that’s causing the child’s symptoms… or I don’t think I’m going to treat it, I don’t tell the parents that … cause… they would expect that you’re going to give them something for that, so I usually say it’s normal, just a bit congested or something.” GP5*

#### Theme 4: Conflicting management advice between PCPs

While the lack of evidence and possible side effects and sedation were the reasons some PCPs cautioned the use of OTC medications for young children, for other PCPs, it was more about the fact that they were no longer recommended for children under the age of 6 due to regulations. GPs commented that they generally discussed with parents that these medications lacked evidence, were a waste of money and had possible side effects in contrast to the minimal benefit they might provide. Pharmacists reported that their strategy was simply to comment that these products were no longer recommended for children under 6, rather than citing a lack of evidence for their use. One pharmacist even queried the reasoning behind the recommendation:

*“I guess the thing with that then is, why do they have the same ingredients for kids over 6s…?” PH1*

When asked, PCPs would make specific recommendations to families about when children could resume normal activities, recognizing that parents would require time off work while their child was deemed “sick”. There was tremendous variability in terms of this advice; some PCPs advised to keep the child home until completely asymptomatic, while others recommended resuming normal activities if the child felt well enough to attend.

*“… if their child is having a minor cold, they are going to school or child care, or kinder…. it’s just going to… spread…” PN2*

There was even disagreement among PCPs, where a couple of PCPs advised parents to keep their child at home but would themselves send their own child back to childcare in the same circumstance due to the pressure of having to go back to work.

*“I do send them if they’ve got a snotty nose, as long as they don’t have a temperature… As a parent, if they don’t have a temperature, they go to school or childcare…As a GP? If they have a snotty nose, hmm probably not recommended!” GP8*

### Motivation: Belief about consequences, professional role and identity, emotion

#### Theme 5: Factors influencing GPs’ advice regarding antibiotic prescribing

In terms of antibiotic prescribing, GPs were mostly reluctant to prescribe unnecessarily—i.e., in situations where they believed the illness was viral and uncomplicated. However, extra-clinical factors such as perception of what they thought parents wanted, parental pressure, and concerns that parents would seek antibiotics elsewhere influenced antibiotic prescribing.

GPs also commented that they were sometimes guided by parents in prescribing antibiotics. Some GPs had the perception that parents expected antibiotics as a treatment for RTI even before coming in for the consultation. Some GPs succumbed to parental pressure if parents were absolutely insistent, concerned that if they didnot prescribe, parents would go elsewhere to obtain a prescription from another doctor. As they want to ‘‘please’’ the parents, it was ‘‘easier to write a script.’’

*“… if they say ‘‘I do want antibiotics’’, unless it’s really late in the day, and I’m really tired and I’ve had enough, I will try and explain why…” GP2*

*“Sometimes they are quite demanding for antibiotics. Then probably you’ve got less threshold … cos you’ve got to make them happy… you try and explain to them the pros and cons of antibiotics, but in the end, you just have to please them…” GP8*

*“…if they are absolutely insistent and I know they are pretty much going to walk out of the door and request for another doctor, I’d give them a script…” GP13*

Delayed prescribing was often mentioned as a strategy for GPs to deal with demanding parents who wanted antibiotics for viral RTIs. Of the 20 GPs interviewed, 18 said they have previously provided antibiotic scripts to anxious parents but cautioned the parents not to start until it was necessary.

*“… the ones who do sort of push, sometimes I leave an open gap, I explain to them, look, I’ll give you a script, but see how the child goes over the next two or three days, if things aren’t getting better… then you can try it, may help, may not.” GP14*

*“… sometimes parents feel happier that they have the prescription because we’re not always open on the weekends, they feel comfortable to have that prescription… instead of going and waiting in the emergency…” GP16*

Although GPs were the only PCPs in this study that could prescribe antibiotics, they were not the only health professionals parents would ask for advice regarding antibiotics. While pharmacists discussed the aetiology of the common cold and the use of antibiotics with anxious parents, MCHN said they would advise parents to query their GPs as to why antibiotics were prescribed in the first place.

*“So I say to them, that if the GP does prescribe you antibiotics, you need to ask the GP, “Why has my child got antibiotics?’” MCHN1*

However, there appears to be a hierarchy where GPs have the ‘‘final say’’ on the management and treatment of RTIs in young children. While PNs, MCHNs and pharmacists provided specific advice such as symptomatic management of RTIs, OTC medication and sometimes even antibiotics, they commented that they would always advise parents to see a GP if the symptoms were severe and/or worse after a couple of days.

## Discussion

### Main findings

Our study applied a systematic approach using the theoretical domain framework (TDF) and Capability, Opportunity and Motivation (COM-B) model to explore PCPs’ attitudes and practice in managing RTIs in young children. From the qualitative interviews, it appears that the management of RTIs is a consultation process involving PCPs and parents of the sick child, however, many extra-clinical factors such as time constraints on PCP; parental anxiety; GPs’ perception of what parents want; fear of losing patients; and the perception of parental pressures influenced the management and treatment of RTIs in young children. GPs providing an antibiotic script and asking parents not to fill it for a couple of days was often addressed as a strategy to deal with anxious parents wanting unnecessary antibiotics. Conflicting management advice between PCPs on OTC medication and when a young child should resume normal activities were also seen as a barrier to the management of RTIs in this age group. As the management of RTIs in young children involves combined input from PCPs and parents, education strategies should include all PCPs and parents so that consistent advice is provided to parents to better manage RTIs in young children.

### Interpretation of findings in relation to previously published work

Our study has found that the management of RTIs in young children is a complex interaction consisting of not only symptomatic and/or medical treatment, but also emotional and psychological factors involving decisions from both parents with a sick child and PCPs advising those parents. While guidelines were mostly followed by GPs and other PCPs, many factors were shown to influence the PCPs’ decision making in regards to managing both parents and their sick child.

Our finding that parental anxiety can influence the decision of both PCPs and parents in the management of RTIs in young children has not previously been well documented. Our study showed that GPs perceived a level of anxiety in parents who present with a sick child during consultations and that management was dependent on the level of anxiety and what parents expected from the consultation. While most PCPs tried to reassure the parents that their child was fine and discussed what symptoms to expect from the RTI, parental anxiety (especially with young children) led to unnecessary OTC medication and antibiotics being advised and/or prescribed. Further studies with parents of young children could reveal the degree of anxiety regarding their sick child and the impact that this has on GPs’ decision to prescribe medications including antibiotics.

International studies have shown that parental concerns for the sick child and seeking additional information might be misinterpreted by physicians as pressure to prescribe medication, especially antibiotics.^15, 18, 21^ Our study supported these findings as GPs commented that parents did have an expectation of antibiotics and that GPs did not want to ‘‘disappoint’’ parents. Our study also noted that time was lacking during consultation in order to educate parents regarding the appropriateness of antibiotics for RTIs and that this contributed to unnecessary prescriptions. It has been suggested that better communication between GPs and parents,^21, 22^ education to improve parents’ understanding of RTIs and management options,^10, 23^ and enabling GPs to have sufficient time to consult might reduce the unnecessary prescribing of antibiotics.

Delayed prescribing has been shown to be effective in reducing the rate of antibiotic use by up to half,^24, 25^ and is a strategy that is preferred by both patients and GPs.^26^ GPs like to use this method to please their patients and encourage shared decisions, which can result in patients feeling empowered to make their own decision regarding antibiotic use.^27^ GPs in our study also mentioned that delayed prescribing is an acceptable method to possibly reduce antibiotic use while simultaneously giving parents something so they feel like therapeutic action is being taken for their sick child. While this approach may be favourable to both patients and GPs, it does not educate patients on the rationale of delaying antibiotic prescribing, and may result in a continued pattern of return visits with the expectation of an antibiotic prescription.^19, 28^ Educating parents to look for certain RTI symptoms in their children, as well as understanding the unnecessary use of antibiotics for a common cold might be a better approach than delayed prescribing.

One of the barriers to educating parents that we reported in our study was the lack of consulting time to educate parents on the management of RTIs, especially with decisions regarding the use of antibiotics. It was often easier and more efficient for GPs to write a script. A previous study involving children under 18 years of age with RTIs found no significant difference in time taken for a physician to prescribe antibiotics or not, hence suggesting it did not take longer to *not* prescribe antibiotics in a consultation.^29^ However, this study did not mention whether educating parents was conducted during the consultation for those physicians who did not prescribe antibiotics. GPs in our study mentioned their limited consultation time restrict opportunities to educate parents, it might be possible to involve other PCPs to discuss RTIs management options with parents.

Our study found differences in the management advice given by PCPs, especially in regards to the use of OTC medications, and opinions on when children can resume normal activities. While evidence is lacking on the effectiveness of OTC medication for the treatment of the common cold, especially in young children,^6, 30^ studies have reported that these medications are still being recommended by physicians.^5, 31, 32^ Our study found that most GPs do not recommend OTC cough and cold medications, but parental anxiety and the need to ‘‘do something’’ have led some GPs to deviate from existing guidelines. Perhaps due to the reasons that these medications are no longer recommended to children under that age of six, pharmacists have turned to natural remedies even though there is no evidence to support their effectiveness. Conflicting advice was also given by PCPs’ regarding when to resume normal activities. While most PCPs agreed when a child should go back to childcare/daycare, the pressure of parents having to work could influence PCPs’ advice to send the child back earlier.

This study has demonstrated that consistent advice from all PCPs is needed in order to better manage RTIs in this cohort. As decisions on how to better manage a young child with a common cold involve complex interactions with PCPs and parents, it is imperative that consistent messages are communicated to parents and that communication between parents and all health-care professionals involved in the child’s well-being is improved.^24, 33^

### Strengths and limitations of this study

As far as we are aware, this is the first study to apply the TDF and the COM-B model to explore practice and assess barriers of managing RTIs in young children. We were also able to comprehensively examine the management of RTIs in young children in primary health care by including the views of GPs, PNs, MCHNs and pharmacists. The overwhelming response we received from PCPs (especially GPs) to participate in this study allowed us to interview PCPs in a wider geographic location.

There are a couple of limitations to our study. All PCPs that we interviewed expressed a genuine interest in this topic and wanted to make a difference in the management of RTIs in young children; this may have led to selection bias. Most importantly, we lacked the views of time constrained PCPs who could not participant in this research. Their views and practices in terms of overcoming barriers such as time constrains are important to aid the development of effective intervention strategies that change management habits of PCPs in order to better manage RTIs in young children.

### Implications for future research, policy and practice

In this study, we used the TDF and COM-B model to demonstrate PCPs’ management of RTIs in young children. While guidelines for the management of RTIs were mostly followed, barriers such as parental pressure; PCPs’ perception of what parents want; lack of consultation time; and parental anxiety could affect guideline adherence. By developing the study using the TDF and the COM-B model, we were able to understand the behaviour of PCPs and parents of young children regarding their management of RTIs and identify areas for change. This knowledge will enable us to undertake an informed approach to the future development of interventions that are targeted towards improving the management of RTIs in young children.

## Conclusions

Many extra-clinical factors, including emotional and psychological factors, influence the decision making of PCPs and parents when it comes to a sick child with RTI. This study provided some reasoning behind the extent to which the management of RTIs in young children has diverted from national guidelines. Based on our findings, we believe that a team approach involving other health-care professionals and the delivery of consistent advice is paramount. Consequently, interventions such as team care approach, strategies that focus on improving communications between PCPs and parents, and educating parents regarding common colds and antibiotic usage should be developed to overcome these barriers to improve the management of RTIs in young children.

## Methods

### Theory

**Fig. 1:**
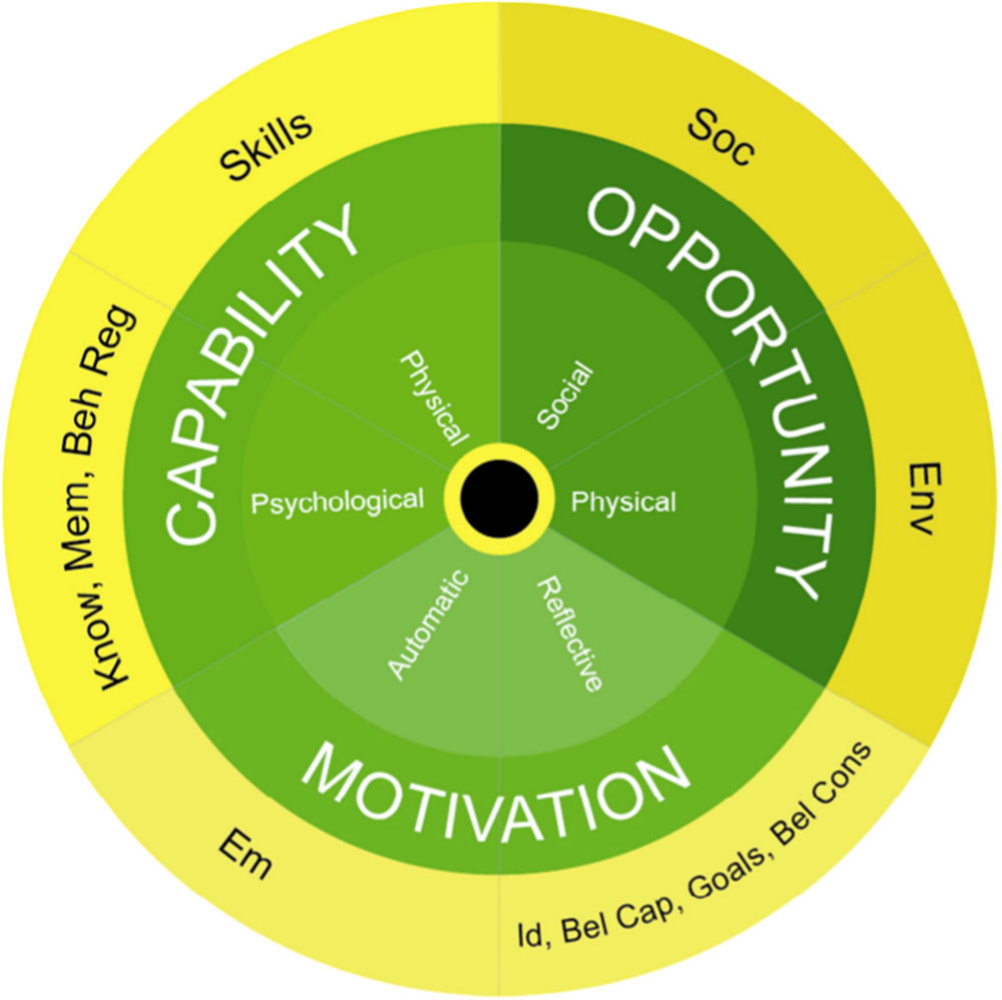
TDF domains linked to COM-B components^48^

In this study, we applied the TDF^34^ and the COM-B model^35^ (Fig. 1) to inform data collection and analysis.

A behavioural theory approach can be used to inform the development of complex interventions by identifying key concepts that will lead to behaviour change and providing a means to select appropriate interventions to behaviour change.^34, 36–38^ Data can then be gathered and accumulated across different contexts, populations and behaviours to provide a comparable evidence base where the different barriers and facilitators to the design and uptake of an intervention can be assessed. This can inform researchers about the effectiveness of interventions and guide future research to refine and develop better interventions. The TDF was developed to simplify and integrate behaviour change theories into a set of 14 theoretical domains derived from 128 constructs from 33 health and social psychology theories that assists with the understanding of behaviour change.^34–39^ The 14 domains consist of knowledge, skills, social/professional role and identity, beliefs about capabilities, optimism, reinforcement, intentions, goals, memory, attention and decision processes, environmental context and resources, social influences, emotion, and behavioural regulation.^39^ The TDF has been used to design research studies such as improving hand hygiene compliance,^40^ treatment pathways,^41, 42^ forecasting health expectancy,^43^ and improving the uptake of vaccination.^44, 45^

The COM-B model was also developed, as a new framework for a behaviour system (B) using three essential conditions: capability (C), opportunity (O) and motivation (M).^38^ For a given behaviour to exist, these three conditions must be met. There are six components to the COM-B model: physical capability; psychological capability; reflective motivation; automatic motivation; physical opportunity; and social opportunity.This framework helps researchers to understand behaviour and, thereby, characterise and design behaviour change interventions.^35^

### Design

Cross-sectional qualitative research design comprising semi-structured interviews with PCPs.

### Recruitment

PCPs were recruited across metropolitan Melbourne to participate in this study. The contact details of GPs and PNs were generated from an existing general practice database at Monash University (Melbourne, Australia), while the contact details for MCHNs and pharmacists were obtained via the Maternal Child Health Services website^46^ and the local business directory, respectively. Invitation letters and research project explanatory statements were sent to the practices, including the contact details of the researcher. The study was also advertised via a local primary health network in the south east region of Melbourne to facilitate recruitment. We included PCPs who see at least five children under 5 years of age per week. Recruitment was limited to one PCP per practice.

### Procedure

The interview questions were developed from a literature review and the TDF to identify the barriers and enablers of current practice aimed at preventing and minimising RTIs in young children. The data collected for analysis included questions regarding the diagnosis and management of RTIs in young children, treatment options such as OTC medications and antibiotics, and the appropriate time to return to normal activities after a RTI (Table 1). The interview questions were piloted with two GPs, one PN, one MCHN, and one pharmacist in order to validate the questions (this data was not included in the final analysis).

**Table 1:** Interview schedule using TDF and COM-B to determine themes

COM-B component identified in the behavioural analysis	Domains linking to COM-B component	Interview example questions	Themes
Capability—psychological	Knowledge (an awareness of the existence of something)	What OTC medications do you recommend, if any?	PCPs’ advice on managing RTIs in young children
Capability—physical	Skills (an ability or proficiency acquired through practice)	How do you diagnose the infection?	PCPs’ advice on managing RTIs in young children
		How do you manage the children’s cold symptoms?	
		Can you tell me the process of prescribing antibiotics for RTIs in this age group?	
Motivation—reflective	Social/professional role and identity (A coherent set of behaviours and displayed personal qualities of an individual in a social or work setting)	What if the parents still insist on antibiotics?	Factors influencing GPs’ management advice on antibiotic prescribing
	Beliefs about consequences (acceptance of the truth, reality or validity about outcomes of a behaviour in a given situation)	When should you advice parents to send the child back to childcare, other normal activities?	Factors influencing GPs’ management advice on antibiotic prescribing
Motivation—automatic	Emotion (a complex reaction pattern, involving experiential, behavioural and physiological elements; by which the individual attempts to deal with a personally significant matter or event)	How do you handle the situation if parents are insistent in antibiotics?	Parental anxiety affecting PCPs’ advice on treatment and management of RTIs
		Why don't you recommend over the counter medication?	Factors influencing GPs’ management advice on antibiotic prescribing
			Conflicting management advice between PCPs
Opportunity—social	Social influences (those interpersonal processes that can cause individuals to change their thoughts, feelings or behaviours)	Are you guided by parents in terms of prescribing antibiotics?	System barriers leading to lack of adherence to guidelines

Interviews (approximately 1 h long) were conducted between June 2014 and January 2015 in-person by RB at the PCPs’ work place or at a place convenient to the PCP during practice hours. All participants completed the consent form before the commencement of the interview and were reimbursed for their time with a gift voucher (valued at AUD$120) upon the completion of the interview.

### Analysis

Data from the interviews were digitally recorded and transcribed verbatim. They were then analysed using a thematic approach.^47^ Two researchers (R.B. and B.B.) read the first three transcripts independently to generate initial codes and themes, which were then compared and refined until consensus was reached. A further three transcripts were coded, compared and refined. This process was repeated until all transcripts were coded. Emerging themes were further reviewed and refined to ensure precision of data analysis. After consensus was reached, the codes were matched to the domains within the TDF and mapped to the COM-B system, and the themes were generated within the model (Table 1). Data coded under a specific theme appeared across more than one domain in the TDF, but only appeared within one behaviour in the COM-B model. Data was managed using NVivo Ver.10. Study approval was obtained from the Monash University Human Research Ethics Committee (CF14/1384—2014000648).
